# Personalized Prehabilitation Improves Tolerance to Chemotherapy in Patients with Oesophageal Cancer

**DOI:** 10.3390/curroncol30020118

**Published:** 2023-01-24

**Authors:** Grigorios Christodoulidis, Laura J. Halliday, Athina Samara, Neel Bhuva, Won-Ho Edward Park, Krishna Moorthy

**Affiliations:** 1 General Surgery Department, University Hospital of Larisa, 41110 Larisa, Greece; 2 Department of Surgery and Cancer, Imperial College London, London W2 1NS, UK; 3 Mount Vernon Cancer Centre, East and North Hertfordshire NHS Trust, Northwood HA6 2RN, UK; 4 Imperial College Healthcare NHS Trust, London W2 1NS, UK

**Keywords:** gastroesophageal junction cancer, oesophageal cancer, prehabilitation, chemotherapy, radiotherapy

## Abstract

Background: Prehabilitation programmes aim to optimise patients before and after cancer treatment including surgery. Previous studies in surgical patients demonstrate that prehabilitation improves pre-operative fitness and overcomes the negative impact of neoadjuvant chemotherapy on fitness. The aim of this study was to assess the impact of prehabilitation on the tolerance of neoadjuvant chemotherapy in patients with oesophageal cancer. Methods: Patients with oesophageal or gastroesophageal junction (GOJ) cancer from two oncology centres were retrospectively included in the present comparative cohort study; one provided a multimodal prehabilitation programme and one did not offer any prehabilitation. Tolerance of chemotherapy, defined as completion of the full chemotherapy regime as per protocol, was compared between the two groups. Results: In terms of participants, 92 patients were included in this study, 47 patients in the prehabilitation cohort and 45 in the control cohort. Compared with the control group, the prehabilitation group demonstrated an improved rate of chemotherapy completion (*p* = 0.029). In multivariate analysis, participation in prehabilitation was significantly associated with an improved rate of chemotherapy completion. Conclusion: The findings of this exploratory study suggest that prehabilitation is associated with better tolerance for chemotherapy. Further research is needed to establish the long-term impact of prehabilitation on oncological outcomes.

## 1. Introduction

The treatment of esophagogastric (OG) cancers frequently incorporates neo-adjuvant chemotherapy and/or radiotherapy [1,2,3]. Surgery for esophagogastric (OG) cancer includes major operations that are associated with high morbidity and mortality [3,4]. It has been demonstrated that cardiopulmonary fitness and clinical status prior to major surgery are positively related to postoperative outcomes [5,6]. Patients with OG cancer are typically frail with reduced functional capacity and multiple co-morbidities [7,8,9,10], added to which they often receive neoadjuvant chemo(radio)therapy (NAC), which further reduces their functional capacity prior to surgery [11].

Despite the improved efficacy of the newest chemotherapy agents resulting in favourable oncological outcomes, side effects of anti-cancer regimes remain an important concern [12]. Chemotherapy affects multiple systems, such as cardiovascular, digestive and hematopoietic systems, and has a significant impact on the patients’ overall quality of life [12,13]. Moreover, NAC in older, frail patients with OG cancer may have a negative impact on preoperative cardiorespiratory fitness [14], which, in turn, is associated with higher post-operative mortality.

Prehabilitation programmes that include structured exercise interventions prior to surgery have been shown to improve pre-operative fitness and reduce post-operative complications [15,16]. There is increasing evidence highlighting the value of exercise interventions in patients undergoing chemotherapy to prevent the decline in fitness associated with oncological treatment [11,17,18,19,20]. A recent pilot randomised controlled trial demonstrated that oesophageal cancer patients who undergo prehabilitation have a higher completion of chemotherapy protocols as planned [18]. However, it remains to be seen whether these findings will be replicated in other prehabilitation programmes and wider clinical practice. The primary aim of this retrospective case-control study within a real-world clinical setting was to evaluate the impact of prehabilitation on tolerance to chemotherapy in a group of OG cancer patients receiving NAC prior to surgery.

## 2. Materials and Methods

### 2.1. Study Participants

This is a retrospective comparative cohort study of consecutive patients, comparing two specialist OG cancer treatment centres. Ethical approval for retrospective analysis of patient data was granted by the UK Health Research Authority (ref: 268837).

The study includes patients diagnosed with oesophageal and gastroesophageal junctional adenocarcinoma between January 2016 and June 2018 who underwent neoadjuvant treatment followed by surgical resection at two cancer centres. Both centres are located in North West London, United Kingdom. Centre A offered a multi-modal prehabilitation programme, lasting an average of 12 weeks. The details of this programme have been published [17] and are summarized below. Centre B did not offer prehabilitation. 

#### Details of the Prehabilitation Programme at Centre A

In accordance with World Health Organization guidelines, a personalised home-based program was prescribed by an exercise therapist, including a moderate-intensity activity with a minimum duration of 150 min. A tailored exercise programme consisting of aerobic and strength exercises was prescribed based on FITT (frequency, intensity, time and type) principles.

Every week, there was a scheduled telephone touch point with an exercise therapist. When exercise goals were achieved, the exercise programme was progressed according to FITT principles. For those who were unable to meet their goals, the programme was adapted to their clinical condition and re-evaluated at the next touchpoint. Dietetic support was provided by a specialist dietitian who undertook an assessment of nutritional status, including identification and stratification of nutritional risk. A plan was agreed based on symptoms, dietary eating habits and nutritional deficiencies. Weekly or fortnightly phone calls from the dietitian were used to monitor adherence to the programme. Interventions, such as oral supplementation or enteral feeding via a jejunostomy, were established when risk was identified. Psychometric screening was completed for all patients and psychological support was provided by a clinical nurse specialist trained in Level 2 psychological interventions. 

The overall aim was to explore and address anxieties or concerns the patient may have regarding their diagnosis, symptoms and/or treatment plan, facilitate adaptation to their current psychological health and disease state and improve self-efficacy. 

Motivational interviewing techniques were used by all professionals to identify any potential barriers or facilitators to adherence and facilitate positive behaviour change. This was accompanied by a timeline of agreed goals with personalised written and visual information.

The prehabilitation programme started at the point of diagnosis, once a decision to proceed with curative resection had been made, and continued throughout NAC until the time of surgery. All patients at centre A who underwent surgical resection with curative intent were eligible to participate in the prehabilitation program.

Centre B did not provide prehabilitation. There were no other significant differences in pre-operative care, other than the provision of prehabilitation. Dietetic support in centre B is consistent with national guidelines and consists of an initial assessment and identification of risk followed by further interactions only if there is any deterioration in status. 

In both centres, the same chemotherapy and chemo-radiotherapy regimes were used. Patients who required chemotherapy received either 3 cycles each of Epirubicin, Cisplatin and Capecitabine (ECX) or Epirubicin, Oxaliplatin and Capecitabine (EOX) or 4 cycles of Fluorouracil, Leucovorin, Oxaliplatin and Docetaxel (FLOT). Oncologists in both centres attend the weekly specialist multi-disciplinary meeting and work to similar protocols in terms of choice of chemotherapy regimen and clinical behaviours, such as tailoring of the regimen to each individual patient, dose reduction, treatment cessation, etc.

### 2.2. Primary and Secondary Endpoints

The primary outcome of this study was completion of the full chemotherapy/chemoradiotherapy course as per protocol. Secondary outcomes included the identification of factors that affect completion of the full chemotherapy course. Deviations from per-protocol chemotherapy completion included dose reduction, treatment interruption and treatment withdrawal.

Data were collected by an independent researcher (GC) who was not involved in the prehabilitation programme or a part of the oncology team at either centre. The following parameters were recorded for all study participants: age, Karnofsky [21] and American Society of Anaesthesiology (ASA) scores [22], co-morbidities, tumour location, histology and cycles of chemotherapy/chemoradiotherapy that were completed. These were prospectively collected in two electronic datasets, one in each participating institute, and analysed retrospectively for this study. Adverse events that led to deviations from per-protocol chemotherapy completion were recorded retrospectively by reviewing the clinical notes at each centre.

The Karnofsky Performance Scale Index is an assessment tool for functional impairment. It can be used to compare the effectiveness of different therapies and to assess the prognosis in individual patients. In general, a lower Karnofsky score is associated with a decreased survival rate [21].

### 2.3. Statistical Analysis

The results were analysed using GraphPad Prism 8.4.3 for Mac (GraphPad Software, San Diego, CA). Normal distribution of the data was performed by application of the D’Agostino and Pearson Omnibus normality test. Comparisons of continuous variables were performed with two-tailed unpaired t-test for parametric data and Mann–Whitney U-test for non-parametric data. The categorical outcomes were tabulated in 2 × 2 tables and were assessed by performing the Chi square test. Multiple logistic regression analysis was performed to identify prognostic factors regarding the number of patients that did not complete chemoradiotherapy. All the co-variates were entered into the logistic regression model. All data from patients lost in follow-up were excluded from the analysis. Differences were deemed significant with a *p* < 0.05.

## 3. Results

### 3.1. Baseline Characteristics

**Figure 1:**
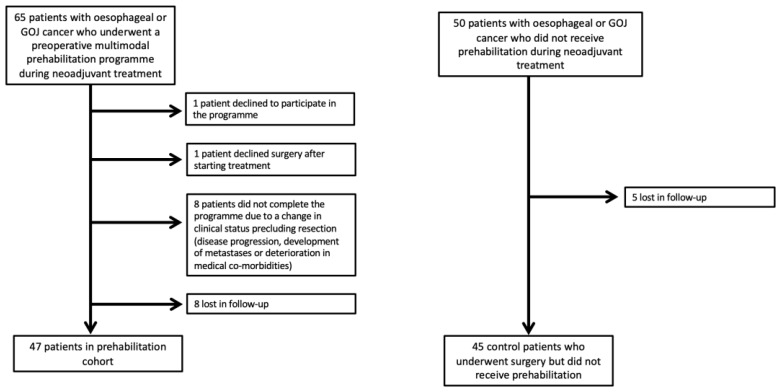
Study participant flow chart.

Figure 1 provides details of the two patient cohorts whose data were included in the study. Thus, 47 patients were included in the prehabilitation group and 45 in the control group went on to have surgery after completion of NAC and were included in the study.

Patients’ baseline characteristics are presented in Table 1. Both groups were comparable in their demographic profile. There was a significant difference in the Karnofsky performance status score between the two groups, with the prehabilitation group having a lower score, but all other clinical and oncological variables were comparable. There were no significant differences in the chemotherapy protocols used in the two groups (Table 1).

Therefore, 44 patients (93.6%) from the prehabilitation group and 35 (77.7%) patients from the control group completed their chemotherapy schemes as per protocol. The difference was statistically significant (*p* = 0.029).

In the prehabilitation group, three patients had their treatment stopped and in the control group, the treatment was stopped in seven patients and the dose reduced in three. There was no difference in between the two groups for adverse events (Table 2), which were severe nausea and vomiting (six patients, 7%), neurological deficits (four patients, 4%), acute renal failure (two patients, 2%) and cardiovascular events (one patient, 1%)

Continuous data are presented as mean ± SD unless otherwise stated. BMI: Body Mass Index; COPD: Chronic Obstructive Pulmonary Disease; ASA: American Society of Anaesthesiologists; SD: Standard Deviation, EOX: Epirubicin, Oxaliplatin and Capecitabine, ECX: Epirubicin, Cisplatin and Capecitabine

### 3.2. Chemotherapy Completion and Multivariate Analysis

From the multiple logistic regression analysis of statistically significant parameters on univariate analysis, participation in prehabilitation was the only factor associated with improved rates in completion of chemotherapy (Table 3). More specifically, prehabilitation changed the factors that were associated in univariate analysis with completion of chemotherapy and included younger age, fewer comorbidities in terms of better ASA and Karnofsky scores.

## 4. Discussion

This study found that prehabilitation has a positive impact on the tolerance of neo-adjuvant chemotherapy in oesophageal cancer patients and is a prognostic factor of successful chemotherapy treatment completion. Previous studies have demonstrated that prehabilitation during neo-adjuvant therapy can preserve cardiorespiratory fitness [18]. The beneficial effect of prehabilitation on the completion rate of neo-adjuvant therapy demonstrates a further potential benefit from starting prehabilitation as soon as the decision for curative treatment is made.

These findings are in keeping with those of Allen et al., who also reported higher completion rates of neo-adjuvant therapy in oesophageal cancer patients who received multimodal prehabilitation [18]. There is significant heterogeneity in the content and design of prehabilitation programmes [19,20] and although both studies used a multimodal approach to prehabilitation and employed a combination of aerobic and strength exercises, the interventions and type of exercises varied between the studies, as did the type of supervision. This demonstrates that the benefit of prehabilitation on completion of neo-adjuvant therapy in oesophageal cancer patients is not confined to a single protocol or programme structure, and these benefits may be replicated in wider clinical practice. Further research is needed to examine the impact of prehabilitation on neoadjuvant therapy completion in other cancer groups.

While it has been shown that prehabilitation programmes improve pre-operative physical, nutritional and psychological status [20] and reduce postoperative mortality and morbidity [21], this study demonstrates that prehabilitation programmes result in better oncological outcomes and improve the tolerability of neo-adjuvant and adjuvant therapies. By increasing treatment completion as per protocol, prehabilitation may play a critical role in prolonging disease-free survival [1,23].

In addition to improved chemotherapy tolerance, the potential benefits of prehabilitation on response to neo-adjuvant therapy have been demonstrated in other cancer diagnoses (reference). Evidence of improved tumour regression was seen in patients with locally advanced rectal cancer who underwent prehabilitation following neo-adjuvant chemoradiotherapy [24]. Tumour vascular remodelling was also evident in both animal models and clinical studies of pancreatic patients who received pre-operative exercise during neo-adjuvant chemotherapy [25]. 

Although the results of this study are promising, further research is needed to explore the potential impact of pre-operative exercise on the histological response to neoadjuvant therapy in oesophageal cancer, including long-term oncological outcomes and other possible factors, that may impact upon survival and recurrence, such as Mandard score and pathological staging.

### Limitations

There are several limitations to this study. This is an observational study and, although the groups were comparable at baseline (except for Karnofsky score), other unmeasured confounding factors may be present. The Karnofsky score was significantly higher in the control group, suggesting a better performance status in the controls than the prehabilitation group. The potential influences of two different institutes as case group and control group were also considered as possible source bias. Despite this, the rates of chemotherapy completion were still higher in the prehabilitation group. The unknown level of recreational exercise in the control group and the absence of adherence for the prehabilitation group are additional limitations of this study. 

Although this study supports the benefits of multi-modal prehabilitation in terms of treatment completion in patients diagnosed with oesophageal and OG cancer, it is limited by the small sample size. For the oncological benefits of prehabilitation to be recognised, further investigation on the impact of prehabilitation on short- and long-term oncological outcomes in different cancer diagnoses is warranted within a large-scale, multi-centre randomised controlled trial.

## Figures and Tables

**Table 1:** Patient baseline characteristics.

Demographics	Prehabilitation Group, *n* = 47	Non-Prehabilitation Group (Control), *n* = 45	*p*-Value
Female, *n* (%)	9 (19%)	7 (16%)	0.649
Age, years	67.6 ± 10.2	65.8 ± 10.2	0.379
BMI, kg/m^2^	26.5 ± 2.0	27.0 ± 5.4	0.82
ASA Class, *n* (%)			0.898
I-II	39 (83%)	38 (84.5%)	
III-IV	8 (17%)	7 (15.5%)	
Karnofsky Score	93.9 ± 10.0	99.3 ± 3.3	0.002
Cardiac Disease, *n* (%)	23 (49%)	17 (37%)	0.28
Respiratory Disease, *n* (%)	10 (21%)	6 (13%)	0.314
Chemotherapy regime, *n* (%)			0.834
EOX	18 (38%)	20 (45%)	
ECX	13 (28%)	11 (24%)	
Other	16 (34%)	14 (31%)	

**Table 2:** Adverse events.

Adverse Event	Prehabilitation (*n* = 3)	Control (*n* = 10)
Nausea and vomiting	2	4
Renal failure	0	2
Neurological	1	3
Cardiovascular	0	1

**Table 3:** Statistical analysis of variables associated with completion of chemotherapy.

Variables	Univariate Analysis	Multiple Logistic Regression Analysis		
	*p*-Value	Odds Ratio (OR)	95% C.I.	*p*-Value
Age	0.02	0.11	(0.0123, 1.0278)	0.101
Histologic Type	0.368	1.03	(0.6725, 1.571)	0.449
ASA score	0.036	1.03	(0.745, 1.428)	0.203
Radiotherapy	0.756	0.43	(0.1310, 1.397)	0.764
Karnofsky score	0.017	0.98	(0.863, 1.110)	0.501
Prehabilitation	0.003	10.93	(1.044, 114.460)	0.046

## Data Availability

Available upon request from corresponding author.
